# Does Routine Endoscopy or Contrast Swallow Study After Esophagectomy and Gastric Tube Reconstruction Change Patient Management?

**DOI:** 10.1007/s11605-016-3268-y

**Published:** 2016-11-14

**Authors:** N. Nederlof, J. de Jonge, T. de Vringer, T. C. K. Tran, M. C. W. Spaander, H. W. Tilanus, B. P. L. Wijnhoven

**Affiliations:** 1 Department of Surgery, Erasmus MC University Medical Centre Rotterdam, ’s-Gravendijkwal 230, 3015 CE Rotterdam, The Netherlands; 2 Department of Gastroenterology and Hepatology, Erasmus MC, University Medical Center, ’s-Gravendijkwal 230, 3015 CE Rotterdam, The Netherlands

**Keywords:** Esophagectomy, Contrast swallow, Endoscopy, Leakage, Anastomosis, Complication

## Abstract

**Background:**

Anastomotic leakage is a severe complication after esophagectomy. The objective was to investigate the diagnostic and predictive value of routine contrast swallow study and endoscopy for the detection of anastomotic dehiscence in patients after esophagectomy.

**Methods:**

All patients who underwent contrast swallow and/or endoscopy within 7 days after oesophagectomy for cancer between January 2005 and December 2009 were selected from an institutional database.

**Results:**

Some 173 patients underwent endoscopy, and 184 patients underwent a contrast swallow study. The sensitivity of endoscopy for anastomotic leakage requiring intervention is 56 %, specificity 41 %, positive predictive value (PPV) 8 %, and negative predictive value (NPV) 95 %. The sensitivity of contrast swallow study for detecting leakage requiring intervention in patients without signs of leakage was 20 %, specificity 20 %, PPV 3 %, and NPV 97 %.

**Conclusions:**

In patients without clinical suspicion of leakage, there is no benefit to perform routine examinations.

## Introduction

Leakage of the cervical esophagogastrostomy after esophagectomy with gastric tube reconstruction occurs in 5–25 % of patients, and is associated with significant morbidity, and accounts for 25–50 % of postoperative deaths.^1^–^3^ Signs and symptoms of anastomotic leakage are fever, tachycardia, and manifestations at the surgical site including redness, swelling, and drainage of saliva and pus. Appropriate local drainage, intravenous antibiotics, and enteric tube feeding or parental nutrition can manage the majority of anastomotic leakages conservatively. Sometimes, surgical or radiological intervention may be required. In order to detect anastomotic leakage before clinical signs develop and the patients deteriorate, contrast swallow and/or endoscopy are often performed within the first week after surgery. However, it has been reported that contrast swallow studies have a low sensitivity and specificity, failing to contribute to clinical decision making. There is also a risk of aspiration leading to pulmonary complications.^4^–^8^ Upper gastrointestinal (GI) endoscopy has the advantage of direct visualization and quantification of dehiscence, necrosis, or ulcers, and it may be performed in patients who are sedated and intubated.^8^–^11^ On the other hand, there is a fear of iatrogenic injuries and worsening of the anastomotic dehiscence.

The objective of this study is to investigate the diagnostic and predictive value of routine contrast swallow study and endoscopy in the postoperative management of patients with a cervical anastomosis after esophagectomy and gastric tube reconstruction (GTR) for esophageal carcinoma. Our hypothesis was that routine diagnostic studies do not contribute to the early detection of anastomotic leakage.

## Methods

In this retrospective cohort study, all patients who underwent esophagectomy with gastric tube reconstruction and a cervical anastomosis for esophageal cancer at the Erasmus University Medical Center Rotterdam between January 2005 and December 2009 were included. Patient’s demographics, treatment characteristics were retrieved from a prospective, institutional database. This database includes age, sex, medical history, operative approach, site (neck or thorax) and type of anastomosis (end-to-end or end-to-side), and details on postoperative follow-up including complications and their treatment. As part of the postoperative protocol, patients were scheduled for a contrast swallow study and endoscopy 7 days postoperatively.

### Surgical Technique

For tumors at the gastroesophageal junction, a transhiatal esophagectomy was preferred. Tumors of the mid and distal esophagus were resected by a right transthoracic approach. All operations were supervised by one of two specialized senior gastrointestinal surgeons. A gastric tube was created by the aid of a linear stapling device, TLC 55 (Ethicon, Johnson & Johnson, Amersfoort, The Netherlands) or 60 mm GIA (Autosuture, Covidien, Zaltbommel, The Netherlands), making a 3–4 cm-wide tube along the greater curvature of the stomach. During the period the study was done, the neck incision is routinely closed with subcuticular stitch. Drains were not routinely placed. The anastomosis was not reinforced with an omental flap or other vascularized tissues. The cervical anastomosis was created end-to-end (ETE) or end-to-side (ETS) with a running PDS 3/0 suture (Ethicon, Johnson & Johnson, Amersfoort, The Netherlands) depending on the preference of the surgeon or as part of a previously published randomized controlled trial.^12^

### Postoperative Management

Until anastomotic integrity was proven by contrast swallow or endoscopy, patients were fed through a nasojejunal feeding tube with the distal tip situated and fixated in the jejunum and kept nil by mouth. As part of the standardized clinical pathway, a contrast swallow and/or an endoscopy before commencing oral intake were done. This was scheduled around postoperative day 7, but, in some patients, it was delayed due to logistical reasons. When endoscopy and/or contrast swallow confirmed integrity of the anastomosis, or in case of a minor anastomotic dehiscence (dehiscence of less than ¼ of the circumference) without signs of sepsis, oral feeding was gradually resumed, starting with sips of water on the seventh postoperative day.

Treatment of an anastomotic leakage depended on the presence of local and/or systemic manifestations of the leakage. In all patients, the cervical wound was opened for drainage. In patients with a mediastinal abscess, antibiotic treatment with percutaneous drainage was performed. In case of circular necrosis of the conduit, surgical treatment such as a revision of the anastomosis or takedown under general anesthesia was indicated.

### Contrast Swallow

Contrast swallow studies were performed using visipaque water-soluble contrast media (Visipaque^TM^ Iodixanol, GE Healthcare). The patient was instructed to swallow 200 mL of contrast fluid while the attending radiographer made the X-rays from three different positions (anterior-posterior, lateral, and 270 degrees). The radiologist reported on the findings of the study with the attending surgeon at the day of the examination. Some patients received prophylactic antibiotic treatment when aspiration occurred, based on clinical judgment.

### Upper Gastrointestinal Endoscopy

A trained specialist according to the hospital’s protocol performed endoscopy. If requested, patients were sedated. A gastrointestinal videoscope was introduced to assess the integrity and aspect of the esophagogastric anastomosis and gastric tube by the attending consultant gastroenterologist.

### Definitions of Anastomotic Leakage

A clinical leak was defined according to Bruce et al. 13 by “drainage of saliva or gastrointestinal content from the surgical join between the esophagus and gastric tube. Contents may emerge either through the wound or at the wound site, or may be collected near the anastomosis with or without systemic complications. Clinical leakage was defined as presence of luminal contents through the drain or wound site causing local inflammation, e.g., fever (temperature > 38.0 °C) or leukocytosis (white cell count > 10,000/l).” Radiological leakage was defined as extra luminal contrast on contrast swallow study not due to aspiration, as judged by the attending radiologist. For endoscopy, leakage was defined as a partial or complete dehiscence of the esophagogastric anastomosis. Local necrosis, ischemia, and ulcers without a visible dehiscence defined preliminary signs and considered as an abnormal endoscopy. The gastroenterologist and radiologist were not aware about the results of the contrast swallow or endoscopy, whichever was done first.

The contrast swallow or endoscopy was considered true positive when the test showed anastomotic dehiscence, and patients developed a clinical leak (grades I–IV according to Clavien Dindo). When normal oral intake was started and patients did not show signs of a clinical or endoscopic leak, the modality was considered false positive. The study modality was considered false negative if, despite normal swallow and/or endoscopy, patients developed clinical signs of leakage. It was considered true negative when patients did not develop a clinical leak. Leakage originating from the blind end of the gastric tube in patients with an end-to-side anastomosis was also considered as anastomotic leakage. An intervention was defined as all surgical and radiological interventions for anastomotic leakage (Clavien-Dindo grade I or higher) including opening the neck wound at bedside.

### Comprehensive Complication Index

Each postoperative event in each patient was assessed and graded according to the Clavien-Dindo classification. The Comprehensive Complication Index (CCI) is calculated as the sum of all complications that are weighted for their severity by patients and physicians. The final formula yields a score from 0 (no complication) to 100 (death). It summarizes the entire postoperative experience of the patient with respect to complications.

### Statistical Analysis

Values are shown as means and standard deviation (SD) or as medians with their inter-quartile range, as appropriate. Groups were compared using non-parametrical Mann–Whitney *U* test or Student’s *t* test, if normally distributed. For cross tabulations, Pearson’s Chi Square test with continuity correction was used. All statistical analyses were performed on the statistical package SPSS 20.0 (SPSS Inc, Chicago, Illinois, USA). A *P* value < 0.05 was considered statistically significant. To rule out systematic differences between groups, a logistic regression method was used to compare groups regarding the prevalence of leakage as well as other contributors, such as gender, neoadjuvant treatment, anastomosis, surgical approach and comorbidity, and radical resection.

## Results

Between January 1, 2005 and December 31, 2009, 308 patients underwent esophagectomy with gastric tube reconstruction and a cervical anastomosis. Some 173 patients underwent upper endoscopy, 184 patients underwent a contrast swallow, and 95 patients had both examinations done. The median (range) time between the operation and diagnostic test was 7 6–12 days. Reasons for delay beyond postoperative day 7 of the contrast swallow or endoscopy were leakage already clinically evident, patient on the ventilator in ICU, logistic reasons, and patients were too sick to undergo an endoscopy or contrast swallow. The logistic regression test performed to compare equality between patient populations in the two groups had a log likelihood of 319,417, no statistical differences were found between groups.

### Endoscopy

**Fig. 1:**
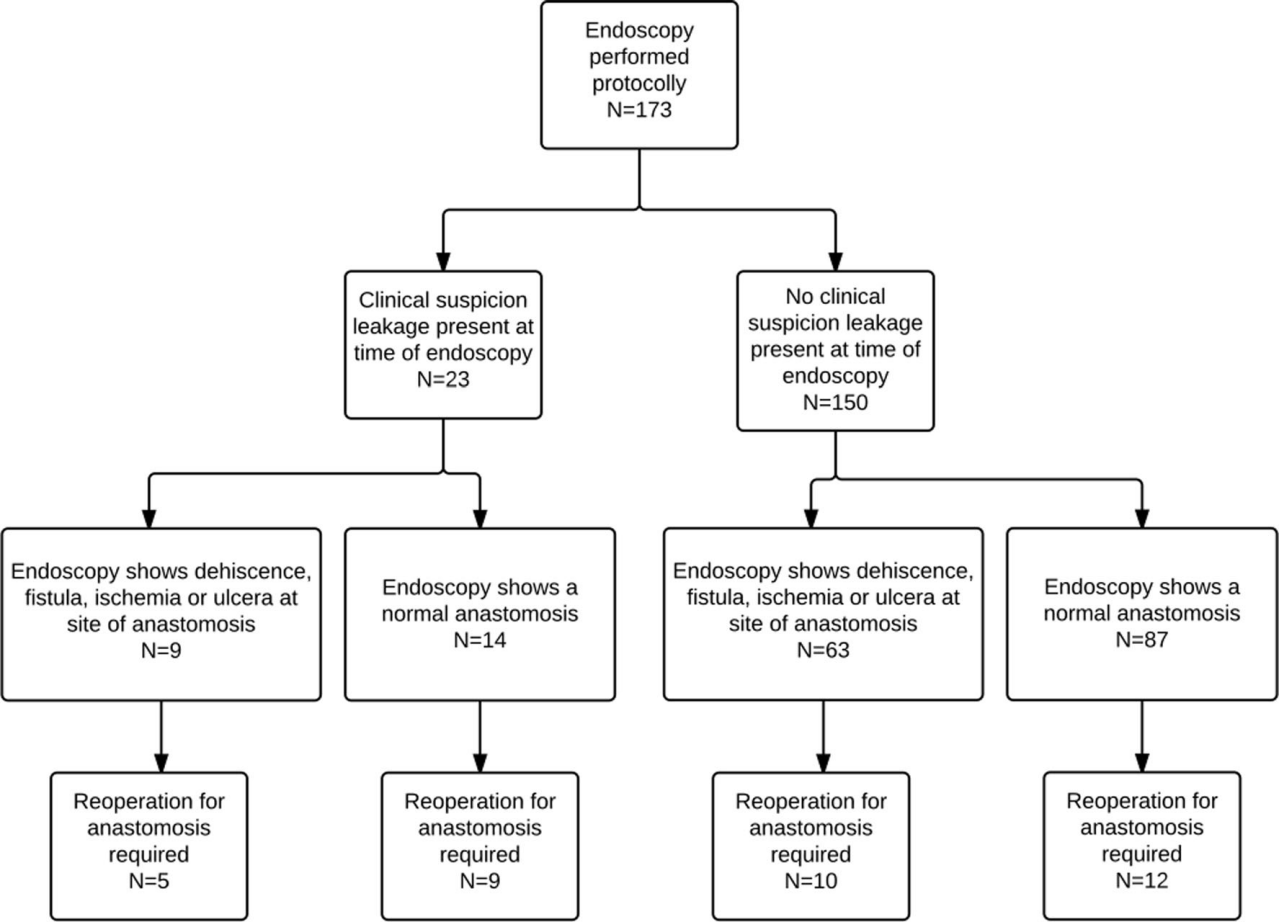
Flowchart of patients; routine endoscopy

Clinical signs of anastomotic leakage were present in 23 of 173 patients (13 %). In 14 of 23 patients (61 %), anastomotic dehiscence, ischemia of the gastric tube, ulcers, and/or necrosis were confirmed by endoscopy, and 9 patients (64 %) required a reoperation (take down of the anastomosis (*n* = 1) and revision of the anastomosis (*n* = 5), opening neck wound (*n* = 3)). In 9 patients, a normal anastomosis was seen by endoscopy. Of the 9 patients with a normal endoscopy, 5 patients required an intervention (revision of the anastomosis (*n* = 1), drainage of mediastinal abscess (*n* = 1), opening neck wound (*n* = 3)) (Fig. 1).

In 63 of 150 patients (42 %) without a suspicion for leakage, an abnormal endoscopy was reported and 10 patients (16 %) developed a clinical leak requiring an intervention (revision (*n* = 2) or takedown of the anastomosis (*n* = 3), or opening neck wound (*n* = 5)) (Fig. 1). In 87 patients, endoscopy showed a normal anastomosis but 12 patients (14 %) required an intervention at a later time point for a clinical leak (stent placement (*n* = 2), disconnection of the anastomosis (*n* = 1), revision of the anastomosis (*n* = 1), opening neck wound (*n* = 8)).

The sensitivity of endoscopy for detecting leakage (requiring intervention) in patients without clinical leakage is 45 %, specificity 41 %, positive predictive value 16 %, and negative predictive value 86 % (Table 1).

**Table 1:** Endoscopy

	Leakage +	Leakage −	
Endoscopy +	10 (7 %)	53 (35 %)	63 (42 %)
Endoscopy −	12 (8)	75 (50 %)	87 (58 %)
	*22* (15 %)	128 (85 %)	150

### Contrast Swallow

**Fig. 2:**
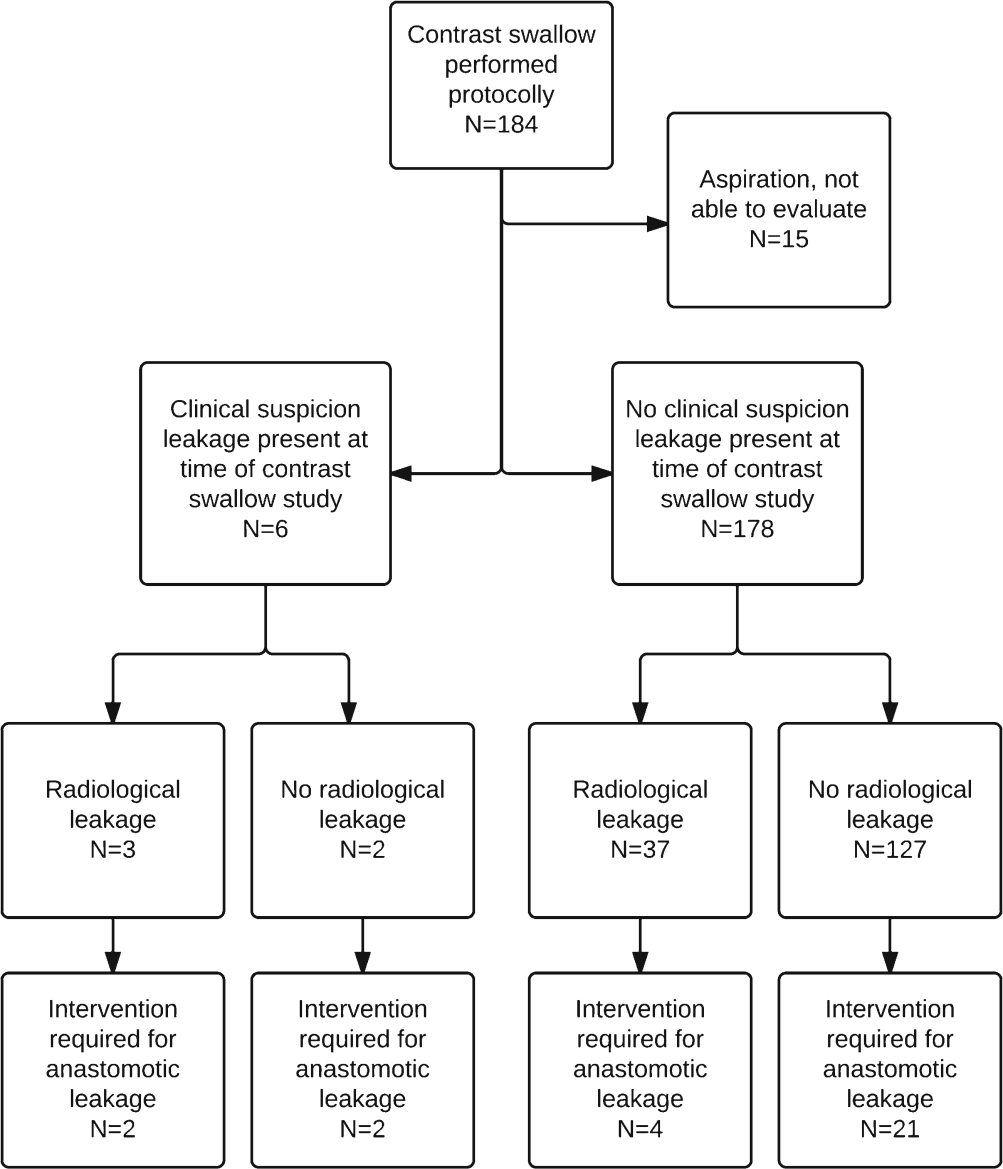
Flowchart of patients; routine contrast swallow examination

In 15 patients, the contrast swallow study could not be evaluated due to aspiration during the study. Therefore, these 15 patients were excluded from the analysis. In 6 of 169 patients (4 %), clinical leakage was present at time of the contrast swallow study and this was confirmed with contrast swallow in 3 patients (Fig. 2). Two patients required percutaneous CT-guided drainage of an abscess caused by anastomotic leakage.

In 37 of 163 patients (23 %) without clinical leakage, radiological leakage was diagnosed by contrast swallow study. One of these patients required underwent endoscopic stenting of the anastomosis. Some 127 patients (78 %) had no leakage on contrast swallow study, and 21 patients (17 %) required an intervention (revision (*n* = 2) or takedown of the anastomosis (*n* = 2), opening neck wound (*n* = 17).

The sensitivity of the contrast swallow study for detecting leakage requiring intervention in patients without signs of leakage was 16 %, specificity 23 %, PPV 11 %, and NPV 84 % (Table 2).

**Table 2:** Contrast swallow study

	Leakage +	Leakage −	
Contrast swallow +	4 (2 %)	33 (12 %)	37 (22 %)
Contrast swallow −	21 (12 %)	111 (67 %)	132 (78 %)
	25 (33 %)	144 (67 %)	169

Table 3 shows the incidence of tumor characteristics and postoperative complications divided into “leakage” and “no leakage” groups for endoscopy, Table 4 for contrast swallow study.

**Table 3:** Endoscopy group; details (*n* = 173)

	Leakage (*n* = 36)	No leakage (*n* = 137)	*p* value
Age (year) median [range]			
Sex (M:F)	29:7	104:33	0.66
Histology			0.65
Squamous cell carcinoma	7 (19 %)	32 (23 %)	
Adenocarcinoma	28 (77 %)	104 (76 %)	
No malignancy after neoadjuvant treatment	1 (4 %)	3 (1 %)	
Tumor site			0.08
Esophagus	33 (92 %)	115 (85 %)	
Gastroesophageal junction	0 (%)	15 (11 %)	
Gastric cardia	3 (8 %)	7 (4 %)	
Tumor stage			0.27
0	1 (4 %)	4 (3 %)	
I	3 (9 %)	11 (8 %)	
IIA	4 (11 %)	35 (26 %)	
IIB	3 (9 %)	10 (7 %)	
III	15 (42 %)	38 (28 %)	
IVA	10 (25 %)	36 (27 %)	
IVB	0 (0 %)	2 (1 %)	
Radical resection (pR0)	26 (72 %)	104 (76 %)	0.73
(Neo) adjuvant treatment	10 (27 %)	43 (32 %)	0.81
Chemoradiation	3 (8 %)	18 (13 %)	
Chemotherapy	7 (19 %)	25 (18 %)	
None			
Comorbidity	17 (47 %)	51 (38 %)	0.82
Cardiovascular	10 (27 %)	25 (18 %)	
Respiratory	3 (9 %)	13 (10 %)	
Diabetes mellitus	1 (2 %)	5 (4 %)	
Malignancy	3 (9 %)	8 (6 %)	
Surgical approach			0.46
Transhiatal esophagectomy	20 (55 %)	88 (65 %)	
Transthoracic esophagectomy	16 (45 %)	49 (35 %)	
Anastomosis			0.26
End-to-end	14 (39 %)	70 (53 %)	
End-to-side	22 (61 %)	65 (47 %)	
Complications	36 (100 %)	102 (%)	0.001
Mediastinitis	18 (50 %)	17 (12 %)	<0.001
Pneumonia	15 (42 %)	49 (36 %)	0.56
Delirium	11 (31 %)	17 (12 %)	0.012
Sepsis	8 (22 %)	6 (4 %)	0.002
Multiorgan failure	4 (11 %)	3 (2 %)	0.035
Vocal cord palsy	1 (3 %)	17 (12 %)	0.13
Bleeding	2 (6 %)	5 (4 %)	0.64
Chyle leakage	1 (3 %)	7 (5 %)	0.69
Respiratory insufficiency	12 (33 %)	16 (12 %)	0.003

**Table 4:** Contrast swallow group, details (*n* = 184) (patients with aspiration during contrast swallow included (*n* = 15))

	Leakage (*n* = 34)	No leakage (*n* = 150)	*p* value
Sex (M:F)	28:6	111:39	0.38
Histology			0.30
Squamous cell carcinoma	2 (6 %)	32 (21 %)	
Adenocarcinoma	31 (91 %)	112 (51 %)	
No malignancy after neoadjuvant treatment	1 (3 %)	6 (18 %)	
Tumor site			0.043
Esophagus	27 (79 %)	114 (76 %)	
Gastroesophageal junction	1 (3 %)	24 (16 %)	
Gastric cardia	6 (18 %)	12 (8 %)	
Tumor Stage			0.12
0	1 (3 %)	7 (5 %)	
I	4 (12 %)	13 (13 %)	
IIA	1 (3 %)	39 (26 %)	
IIB	5 (15 %)	14 (9 %)	
III	12 (35 %)	40 (27 %)	
IVA	11 (32 %)	35 (23 %)	
IVB	0 (0 %)	2 (1 %)	
Radical resection (pR0)	22 (65 %)	119 (81 %)	0.097
(Neo) adjuvant treatment			0.30
Chemoradiation	3 (9 %)	28 (19 %)	
Chemotherapy	9 (26 %)	29 (19 %)	
None	22 (65 %)	93 (62 %)	
Comorbidity			0.26
Cardiovascular	8 (24 %)	29 (19 %)	
Respiratory	1 (3 %)	9 (6 %)	
Diabetes mellitus	2 (6 %)	4 (2 %)	
Malignancy	0 (0 %)	10 (7 %)	
Operating time (mean) SD and range			
Surgical approach			1.0
Transhiatal esophagectomy	23 (%)	101 (%)	
Transthoracic esophagectomy	11 (%)	49 (%)	
Anastomosis			0.87
End-to-end	19 (%)	80 (57 %)	
End-to-side	15 (%)	69 (%)	
Complications	34 (100 %)	95 (%)	<0.001
Mediastinitis	6 (18 %)	5 (3 %)	0.006
Pneumonia	6 (18 %)	42 (28 %)	0.28
Respiratory insufficiency	3 (9 %)	4 (3 %)	0.12
Delirium	3 (9 %)	14 (9 %)	1.0
Sepsis	1 (3 %)	0 (0 %)	0.19
Multiorgan failure	1 (3 %)	1 (1 %)	0.34
Vocal cord palsy	2 (6 %)	17 (11 %)	0.38
Bleeding	1 (3 %)	3 (2 %)	1.0
Chyle leakage	2 (6 %)	5 (3 %)	0.62

#### CCI

All complications of surgery were graded using the Clavien-Dindo classification, and the CCI was calculated for each patient.

In patients without clinical leakage, the median CCI for patients with an abnormal endoscopy was 22.6 (IQR 8.7–44.9). Patients with a normal endoscopy without clinical leakage have a median CCI of 20.9 (IQR 0–26.2). Independently, this difference was statistically significant (*p* = 0.004). In patients without clinical leakage, the median CCI for patients with an abnormal contrast swallow study was 20.9 (0–29.6) as compared to patients with a normal contrast swallow without clinical leakage CCI 8.7 (0–22.6) with *p* = 0.027.

## Discussion

The present study shows that patients without signs or symptoms suggestive of an anastomotic leakage do not benefit from a contrast swallow or upper endoscopy for identifying leaks that require operative or endoscopic interventions. While endoscopy and contrast swallow do show abnormalities in 42 and 20 % of patients, respectively, they do not lead to a change in (conservative) patient management. Hence, aggressive radiological or surgical treatment of patients with abnormal endoscopic findings or contrast swallow does not seem to be indicated. In only a few patients, interventions for anastomotic leakage are needed in due time.

On the other hand, if endoscopy or the contrast swallow study does not show any abnormalities, this does not fully exclude the development of anastomotic leakage and subsequent interventions are needed in 5 and 3 % of patients, respectively (false negative test). The CCI for asymptomatic patients differs significantly for patients without clinical signs of leakage. Even though this difference does not reflect in interventions, it is possible that small, subclinical leakages result in other complications, such as mediastinitis or pneumonia. Furthermore, the CCI is known to be a very sensitive endpoint, as it takes all postoperative complications into account. Still, close surveillance and early recognition of a complication including anastomotic leaks are of utmost importance for best outcomes.

A contrast swallow is the most common routine examination after esophageal surgery. It has several benefits including the low costs and being a relatively safe first-line investigation with a high sensitivity and specificity when interpreted by an experienced radiologist.^14^ However, the disadvantages of aqueous contrast are that it has a low radiographic density and a low mucosal adherence, thus limiting the ability to detect leaks, particularly in case of subtle ones. Boone et al. presented a low sensitivity and positive predictive value in their series of 207 patients and also reported that 53 % of patients already showed clinical signs of leakage. Doerfer et al. produced comparable results and no longer routinely performed a contrast swallow and preferred a CT with contrast. Tanouchi also reviewed a large series (*n* = 331) and found a low sensitivity of routine contrast swallow studies.^5^–^7^ Indeed, in the present study, contrast swallow was reported as normal in 3 patients with clinical suspicion of anastomotic leakage, but CT-guided drainage of a mediastinal abscess was needed in 2 patients at a later point in time. In these patients, CT scanning with oral contrast may be superior to a contrast swallow as it allows detection of peri-anastomotic and mediastinal fluid collections that may need surgical or radiological drainage. Endoscopy is likely to be more useful to assess the severity of anastomotic dehiscence in symptomatic patients. In may help in selecting patients that need surgical revision of the anastomosis including resection of an ischemic segment of the gastric tube. These findings are also supported by Oezcelik 10 and Maïsh.^11^ Schaible 8 and Page et al. 9 presented their retrospective data and concluded that endoscopy is a safe and accurate method to detect early signs of leakage. However, 2 of 8 patients with a normal endoscopy developed a clinical leak that needed a surgical intervention and radiological drainage.

The present study supports the findings of a recent prospective trial that compared the accuracy of contrast swallow, CT with oral contrast and endoscopy for the identification of anastomotic leaks following esophagogastric surgery.^15^ The authors concluded that routine tests of the anastomotic integrity are unnecessary and, when clinical suspicion is high for a anastomotic leak, CT scan is likely the first modality to perform. Our data that supports also another study has shown that flexible upper gastrointestinal endoscopy is more specific in comparison with a contrast swallow study. While it did not improve the identification of clinical anastomotic leakage, it was beneficial to detect gastric necrosis or ulcers and guide management of these patients.

There are several limitations of the present study. Being of retrospective nature, clinical management of anastomotic leaks may have changed over time. Use of self-expandable stents may have led to a decrease of surgical and radiological interventions for anastomotic dehiscence. However, within the time period of the study, the surgical experience has not changed and also the care pathway has remained the same over time. In order to determine the accuracy of diagnostic tests for the assessment of anastomotic leakage, it is of great importance to define the study endpoint in a consistent and unambiguous matter. In the present study, the definition of Bruce et al. was used. However, given the retrospective design of the study, misclassification cannot be ruled out. The Erasmus Medical Centre is a high-volume specialized center for upper GI surgery and radiologists, and gastroenterologists are well trained in the recognition and treatment of postoperative complications. Hence, our data are likely to be externally valid for other specialized centers.
